# Supplementary material for: Degree of Glomerulosclerosis in Procurement Kidney Biopsies from Marginal Donor Kidneys and Their Implications in Predicting Graft Outcomes
Source: J Clin Med. 2020 May 14;9(5):1469. doi: 10.3390/jcm9051469 (PMC7291279; doi:10.3390/jcm9051469)

Table S1 Univariable and multivariable cox regression analyses for post-transplant outcomes between Glomerulosclerosis >20% vs. 11-20%

a) Death-censored graft survival

Factors	Unadjusted		Adjusted	
	HR (95% CI)	P-value	HR (95% CI)	P-value
Glomerulosclerosis >20% vs. 11-20%	0.95 (0.79, 1.14)	0.57	0.95 (0.78, 1.15)	0.59
Donor				
Age	1.01 (0.99, 1.02)	0.37		
Male vs. female	1.02 (0.86, 1.20)	0.84		
Black vs. others	1.41 (1.18, 1.68)	<0.001	1.29 (1.07, 1.56)	0.007
Diabetes vs. others	1.07 (0.91, 1.26)	0.43		
Hypertension vs. others	1.14 (0.91, 1.42)	0.25		
BMI	1.007 (0.997, 1.018)	0.17		
Log serum creatinine	1.24 (1.02, 1.51)	0.03	1.10 (0.89, 1.35)	0.37
DCD vs. others	0.81 (0.57, 1.16)	0.26		
HCV antibody (+ vs. -)	1.44 (0.93, 2.23)	0.10		
Cerebrovascular accident as a cause of death vs. others	1.13 (0.91, 1.40)	0.28		
Machine perfusion (yes vs. no)	0.83 (0.71, 0.98)	0.03	0.85 (0.71, 1.01)	0.06
Recipient				
Age	0.985 (0.976, 0.993)	<0.001	0.99 (0.78, 1.15)	0.59
Male vs. female	1.27 (1.06, 1.51)	0.008	1.32 (1.10, 1.58)	0.003
Black vs. others	1.55 (1.31, 1.82)	<0.001	1.41 (1.18, 1.68)	<0.001
BMI	1.02 (1.00, 1.03)	0.03	1.01 (1.00, 1.03)	0.18
Diabetes vs. others	1.07 (0.91, 1.26)	0.43		
Preemptive vs. others	0.48 (0.33, 0.69)	<0.001	0.51 (0.34, 0.75)	0.001
PRA >60 vs. 0-60%	0.81 (0.53, 1.24)	0.33		
Transplant				
HLA DR mismatch				
0	1.00			
1	0.84 (0.61, 1.16)	0.30		
2	0.79 (0.58, 1.08)	0.14		
Cold ischemic time	1.01 (1.00, 1.02)	0.004	1.01 (1.00, 1.02)	0.01
Transplant period				
2011-2014	1.00			
2008-2010	1.21 (0.98, 1.50)	0.08		
2005-2007	1.14 (0.92, 1.40)	0.22		
Induction therapy				
Thymoglobulin	1.00		1.00	
Alemtuzumab	1.39 (1.09, 1.76)	0.007	1.28 (1.00, 1.63)	0.05
Basiliximab	1.24 (0.98, 1.55)	0.07	1.30 (1.03, 1.64)	0.03

Other induction	1.75 (1.30, 2.36)	<0.001	1.70 (1.25, 2.30)	0.001
No induction	0.91 (0.68, 1.21)	0.51	0.86 (0.64, 1.15)	0.32

CI, confidence interval; HR, hazard ratio; DCD, Donor after cardiac death; HCV, hepatitis C virus; BMI, body mass index; PRA, panel reactive antibody; HLA, human leukocyte antigen

b) All-cause mortality

Factors	Unadjusted		Adjusted	
	HR (95% CI)	P-value	HR (95% CI)	P-value
Glomerulosclerosis >20% vs. 11-20%	0.97 (0.83, 1.13)	0.66	1.00 (0.86, 1.17)	0.98
Donor				
Age	1.02 (1.01, 1.03)	<0.001	1.01 (1.00, 1.02)	0.24
Male vs. female	1.00 (0.86, 1.15)	0.96		
Black vs. others	1.06 (0.90, 1.24)	0.49		
Diabetes vs. others	0.98 (0.84, 1.14)	0.76		
Hypertension vs. others	0.93 (0.78, 1.12)	0.44		
BMI	0.995 (0.985, 1.005)	0.32		
Log serum creatinine	0.96 (0.81, 1.14)	0.65		
DCD vs. others	0.99 (0.74, 1.32)	0.94		
HCV antibody (+ vs. -)	0.53 (0.29, 0.96)	0.04	0.81 (0.44, 1.48)	0.49
Cerebrovascular accident as a cause of death vs. others	1.13 (0.94, 1.37)	0.20		
Machine perfusion (yes vs. no)	0.91 (0.79, 1.05)	0.19		
Recipient				
Age	1.04 (1.03, 1.05)	<0.001	1.04 (1.03, 1.05)	<0.001
Male vs. female	1.14 (0.98, 1.33)	0.08		
Black vs. others	0.92 (0.80, 1.07)	0.29		
BMI	0.999 (0.986, 1.012)	0.87		
Diabetes vs. others	1.52 (1.31, 1.75)	<0.001	1.45 (1.26, 1.68)	<0.001
Preemptive vs. others	0.60 (0.45, 0.80)	<0.001		
PRA >60 vs. 0-60%	0.75 (0.52, 1.09)	0.13		
Transplant				
HLA DR mismatch				
0	1.00			
1	0.91 (0.68, 1.21)	0.51		
2	0.89 (0.68, 1.18)	0.44		
Cold ischemic time	1.001 (0.993, 1.009)	0.81		
Transplant period				
2011-2014	1.00			
2008-2010	1.09 (0.90, 1.33)	0.37		
2005-2007	1.17 (0.97, 1.41)	0.10		
Induction therapy				
Thymoglobulin	1.00		1.00	
Alemtuzumab	1.21 (0.97, 1.50)	0.09	1.39 (1.12, 1.74)	0.003
Basiliximab	1.50 (1.24, 1.82)	<0.001	1.44 (1.18, 1.74)	<0.001
Other induction	1.76 (1.35, 2.28)	<0.001	1.70 (1.31, 2.20)	<0.001
No induction	1.10 (0.88, 1.38)	0.42	1.14 (0.91, 1.42)	0.27

CI, confidence interval; HR, hazard ratio; DCD, Donor after cardiac death; HCV, hepatitis C virus; BMI, body mass index; PRA, panel reactive antibody; HLA, human leukocyte antigen

c) 1-year acute rejection

Factors	Unadjusted		Adjusted	
	OR (95% CI)	P-value	OR (95% CI)	P-value
Glomerulosclerosis >20% vs. 11-20%	1.13 (0.85, 1.50)	0.41	1.10 (0.81, 1.48)	0.54
Donor				
Age	0.993 (0.986, 1.000)	0.06		
Male vs. female	0.81 (0.72, 0.90)	<0.001	0.69 (0.52, 0.92)	0.01
Black vs. others	1.19 (1.06, 1.33)	0.003	1.28 (0.95, 1.73)	0.10
Diabetes vs. others	0.98 (0.87, 1.10)	0.68		
Hypertension vs. others	1.00 (0.88, 1.14)	0.99		
BMI	1.008 (1.001, 1.016)	0.02	1.015 (0.997, 1.032)	0.10
Log serum creatinine	1.06 (0.94, 1.20)	0.32		
DCD vs. others	0.97 (0.81, 1.17)	0.79		
HCV antibody (+ vs. -)	1.34 (1.09, 1.63)	0.005	2.20 (1.09, 4.44)	0.03
Cerebrovascular accident as a cause of death vs. others	1.15 (1.01, 1.31)	0.04	1.61 (1.09, 2.40)	0.02
Machine perfusion (yes vs. no)	1.01 (0.91, 1.13)	0.80		
Recipient				
Age	0.982 (0.976, 0.986)	<0.001	0.988 (0.974, 1.002)	0.08
Male vs. female	1.16 (1.03, 1.29)	0.01	1.55 (1.15, 2.09)	0.004
Black vs. others	1.12 (1.01, 1.25)	0.04	1.02 (0.76, 1.36)	0.89
BMI	0.997 (0.988, 1.007)	0.60		
Diabetes vs. others	0.88 (0.79, 0.97)	0.01	0.70 (0.53, 0.93)	0.01
Preemptive vs. others	0.89 (0.75, 1.06)	0.20		
PRA >60 vs. 0-60%	1.14 (0.94, 1.38)	0.19		
Transplant				
HLA DR mismatch				
0	1.00		1.00	
1	1.17 (0.95, 1.43)	0.13	1.64 (0.83, 3.22)	0.15
2	1.33 (1.09, 1.63)	0.004	2.30 (1.20, 4.42)	0.01
Cold ischemic time	1.006 (1.000, 1.011)	0.05		
Transplant period				
2011-2014	1.00		1.00	
2008-2010	1.05 (0.92, 1.20)	0.44	1.05 (0.75, 1.46)	0.79
2005-2007	1.40 (1.23, 1.59)	<0.001	1.52 (1.08, 2.14)	0.02
Induction therapy				
Thymoglobulin	1.00		1.00	
Alemtuzumab	1.03 (0.88, 1.22)	0.69	1.30 (0.86, 1.95)	0.21
Basiliximab	1.52 (1.32, 1.75)	<0.001	1.68 (1.18, 2.41)	0.004
Other induction	0.96 (0.77, 1.19)	0.70	1.51 (0.90, 2.55)	0.12
No induction	1.14 (0.98, 1.33)	0.10	1.24 (0.81, 1.92)	0.32

CI, confidence interval; HR, hazard ratio; DCD, Donor after cardiac death; HCV, hepatitis C virus; BMI, body mass index; PRA, panel reactive antibody; HLA, human leukocyte antigen

d) Delayed graft function

Factors	Unadjusted		Adjusted	
	OR (95% CI)	P-value	OR (95% CI)	P-value
Glomerulosclerosis >20% vs. 11-20%	0.86 (0.69, 1.07)	0.17	0.82 (0.65, 1.05)	0.11
Donor				
Age	0.981 (0.975, 0.986)	<0.001	0.999 (0.981, 1.017)	0.87
Male vs. female	1.30 (1.20, 1.41)	<0.001	1.46 (1.16, 1.85)	0.001
Black vs. others	1.04 (0.96, 1.14)	0.34		
Diabetes vs. others	1.04 (0.95, 1.13)	0.38		
Hypertension vs. others	1.16 (1.05, 1.28)	0.003	0.82 (0.61, 1.10)	0.19
BMI	1.023 (1.017, 1.029)	<0.001	1.020 (1.005, 1.036)	0.008
Log serum creatinine	1.71 (1.56, 1.88)	<0.001	1.45 (1.08, 1.94)	0.02
DCD vs. others	2.36 (2.07, 2.69)	<0.001	3.95 (2.53, 6.18)	<0.001
HCV antibody (+ vs. -)	1.08 (0.92, 1.27)	0.36		
Cerebrovascular accident as a cause of death vs. others	0.87 (0.79, 0.95)	0.003	0.96 (0.73, 1.25)	0.74
Machine perfusion (yes vs. no)	0.73 (0.68, 0.79)	<0.001	0.50 (0.40, 0.62)	<0.001
Recipient				
Age	0.992 (0.988, 0.996)	<0.001	0.995 (0.984, 1.007)	0.43
Male vs. female	1.36 (1.26, 1.48)	<0.001	1.58 (1.26, 1.98)	<0.001
Black vs. others	1.55 (1.43, 1.68)	<0.001	1.44 (1.15, 1.81)	0.001
BMI	1.027 (1.020, 1.034)	<0.001	1.020 (1.005, 1.036)	0.008
Diabetes vs. others	1.38 (1.27, 1.49)	<0.001	1.23 (0.99, 1.53)	0.06
Preemptive vs. others	0.27 (0.22, 0.32)	<0.001	0.16 (0.09, 0.28)	<0.001
PRA >60 vs. 0-60%	1.04 (0.90, 1.21)	0.59		
Transplant				
HLA DR mismatch				
0	1.00			
1	1.01 (0.88, 1.17)	0.87		
2	1.05 (0.91, 1.20)	0.53		
Cold ischemic time	1.013 (1.009, 1.017)	<0.001	1.014 (1.003, 1.026)	0.02
Transplant period				
2011-2014	1.00			
2008-2010	0.95 (0.86, 1.05)	0.32		
2005-2007	1.03 (0.93, 1.13)	0.59		
Induction therapy				
Thymoglobulin	1.00		1.00	
Alemtuzumab	0.81 (0.71, 0.91)	0.001	0.87 (0.63, 1.21)	0.41
Basiliximab	0.99 (0.89, 1.10)	0.80	1.22 (0.91, 1.62)	0.18
Other induction	1.51 (1.30, 1.74)	<0.001	1.92 (1.27, 2.90)	0.002
No induction	0.96 (0.85, 1.07)	0.44	1.25 (0.88, 1.77)	0.22

CI, confidence interval; HR, hazard ratio; DCD, Donor after cardiac death; HCV, hepatitis C virus; BMI, body mass index; PRA, panel reactive antibody; HLA, human leukocyte antigen

Table S2. Univariable and multivariable cox regression analyses for post-transplant outcomes between Glomerulosclerosis >10% vs. 0-10%

a) Death-censored graft survival

Factors	Unadjusted		Adjusted	
	HR (95% CI)	P-value	HR (95% CI)	P-value
Glomerulosclerosis >10% vs. 0-10%	1.24 (1.13, 1.36)	<0.001	1.27 (1.15, 1.40)	<0.001
Donor				
Age	0.997 (0.991, 1.002)	0.22	-	
Male vs. female	1.00 (0.93, 1.08)	0.95	-	
Black vs. others	1.25 (1.15, 1.35)	<0.001	1.12 (1.02, 1.22)	0.01
Diabetes vs. others	1.18 (1.09, 1.28)	<0.001	1.16 (1.06, 1.27)	0.001
Hypertension vs. others	1.09 (0.99, 1.20)	0.07	-	
BMI	1.007 (1.002, 1.012)	0.01	1.002 (0.997, 1.008)	0.43
Log serum creatinine	1.22 (1.12, 1.33)	<0.001	1.17 (1.06, 1.28)	0.001
DCD vs. others	0.95 (0.83, 1.09)	0.49	-	
HCV antibody (+ vs. -)	1.54 (1.34, 1.78)	<0.001	1.31 (1.12, 1.53)	0.001
Cerebrovascular accident as a cause of death vs. others	1.03 (0.94, 1.13)	0.55	-	
Machine perfusion (yes vs. no)	0.95 (0.88, 1.02)	0.19	-	
Recipient				
Age	0.985 (0.982, 0.989)	<0.001	0.991 (0.988, 0.995)	<0.001
Male vs. female	1.18 (1.09, 1.27)	<0.001	1.16 (1.07, 1.26)	<0.001
Black vs. others	1.53 (1.42, 1.65)	<0.001	1.35 (1.25, 1.47)	<0.001
BMI	1.02 (1.01, 1.03)	<0.001	1.02 (1.01, 1.03)	<0.001
Diabetes vs. others	1.20 (1.11, 1.29)	<0.001	1.16 (1.07, 1.25)	<0.001
Preemptive vs. others	0.67 (0.58, 0.77)	<0.001	0.72 (0.62, 0.83)	<0.001
PRA >60 vs. 0-60%	1.04 (0.89, 1.22)	0.63	-	
Transplant				
HLA DR mismatch			-	
0	1.00			
1	1.12 (0.96, 1.29)	0.15		
2	1.10 (0.95, 1.27)	0.22		
Cold ischemic time	1.007 (1.003, 1.011)	<0.001	1.005 (1.001, 1.009)	0.01
Transplant period				
2011-2014	1.00		1.00	
2008-2010	1.27 (1.15, 1.40)	<0.001	1.25 (1.13, 1.38)	<0.001
2005-2007	1.37 (1.23, 1.51)	<0.001	1.40 (1.26, 1.56)	<0.001
Induction therapy				
Thymoglobulin	1.00		1.00	
Alemtuzumab	1.22 (1.09, 1.36)	<0.001	1.22 (1.09, 1.37)	0.001
Basiliximab	1.02 (0.91, 1.14)	0.76	1.06 (0.95, 1.19)	0.29

Other induction	1.54 (1.35, 1.76)	<0.001	1.46 (1.27, 1.68)	<0.001
No induction	1.08 (0.96, 1.20)	0.19	1.05 (0.93, 1.17)	0.43

CI, confidence interval; HR, hazard ratio; DCD, Donor after cardiac death; HCV, hepatitis C virus; BMI, body mass index; PRA, panel reactive antibody; HLA, human leukocyte antigen

b) All-cause mortality

Factors	Unadjusted		Adjusted	
	HR (95% CI)	P-value	HR (95% CI)	P-value
Glomerulosclerosis >10% vs. 0-10%	1.07 (0.99, 1.16)	0.10	1.03 (0.96, 1.12)	0.40
Donor				
Age	1.018 (1.014, 1.023)	<0.001	-	
Male vs. female	1.01 (0.95, 1.07)	0.83	-	
Black vs. others	0.89 (0.83, 0.96)	0.001	1.02 (0.96, 1.10)	0.51
Diabetes vs. others	1.02 (0.95, 1.09)	0.63	-	
Hypertension vs. others	0.98 (0.91, 1.06)	0.60	-	
BMI	0.999 (0.995, 1.004)	0.68	-	
Log serum creatinine	0.99 (0.92, 1.07)	0.85	-	
DCD vs. others	0.94 (0.84, 1.05)	0.28	-	
HCV antibody (+ vs. -)	1.06 (0.92, 1.21)	0.41	-	
Cerebrovascular accident as a cause of death vs. others	1.01 (0.94, 1.09)	0.78	-	
Machine perfusion (yes vs. no)	0.95 (0.89, 1.01)	0.09	-	
Recipient				
Age	1.04 (1.03, 1.05)	<0.001	1.044 (1.040, 1.048)	0.40
Male vs. female	1.21 (1.13, 1.29)	<0.001	1.13 (1.06, 1.21)	<0.001
Black vs. others	0.87 (0.82, 0.93)	<0.001	1.00 (0.93, 1.06)	0.89
BMI	1.005 (1.000, 1.011)	0.07	-	
Diabetes vs. others	1.51 (1.42, 1.61)	<0.001	1.49 (1.40, 1.58)	<0.001
Preemptive vs. others	0.68 (0.60, 0.76)	<0.001	0.65 (0.58, 0.73)	<0.001
PRA >60 vs. 0-60%	0.92 (0.80, 1.05)	0.21	-	
Transplant				
HLA DR mismatch			-	
0	1.00			
1	1.06 (0.94, 1.19)	0.38		
2	1.06 (0.94, 1.19)	0.37		
Cold ischemic time	1.000 (0.996, 1.003)	0.80	-	
Transplant period				
2011-2014	1.00		1.00	
2008-2010	1.12 (1.02, 1.22)	0.01	1.17 (1.07, 1.27)	<0.001
2005-2007	1.09 (1.00, 1.19)	0.04	1.27 (1.16, 1.39)	<0.001
Induction therapy				
Thymoglobulin	1.00		1.00	
Alemtuzumab	0.98 (0.89, 1.08)	0.64	1.08 (0.97, 1.19)	0.15
Basiliximab	1.26 (1.16, 1.37)	<0.001	1.21 (1.12, 1.32)	<0.001
Other induction	1.27 (1.13, 1.42)	<0.001	1.26 (1.12, 1.42)	<0.001
No induction	0.99 (0.90, 1.08)	0.81	0.99 (0.90, 1.08)	0.80

CI, confidence interval; HR, hazard ratio; DCD, Donor after cardiac death; HCV, hepatitis C virus; BMI, body mass index; PRA, panel reactive antibody; HLA, human leukocyte antigen

c) 1-year rejection

Factors	Unadjusted		Adjusted	
	OR (95% CI)	P-value	OR (95% CI)	P-value
Glomerulosclerosis >10% vs. 0-10%	1.10 (0.95, 1.28)	0.19	1.13 (0.97, 1.31)	0.11
Donor				
Age	0.993 (0.986, 1.000)	0.06	-	
Male vs. female	0.81 (0.72, 0.90)	<0.001	0.81 (0.69, 0.95)	0.009
Black vs. others	1.19 (1.06, 1.33)	0.003	1.14 (0.99, 1.30)	0.06
Diabetes vs. others	0.98 (0.87, 1.10)	0.68	-	
Hypertension vs. others	1.001 (0.879, 1.139)	0.99	-	
BMI	1.008 (1.001, 1.016)	0.02	1.008 (0.999, 1.016)	0.07
Log serum creatinine	1.06 (0.94, 1.20)	0.32	-	
DCD vs. others	0.97 (0.81, 1.17)	0.79	-	
HCV antibody (+ vs. -)	1.34 (1.09, 1.63)	0.005	1.32 (1.03, 1.69)	0.03
Cerebrovascular accident as a cause of death vs. others	1.15 (1.01, 1.31)	0.04	1.04 (0.90, 1.21)	0.57
Machine perfusion (yes vs. no)	1.01 (0.91, 1.13)	0.80	-	
Recipient				
Age	0.982 (0.976, 0.986)	<0.001	0.985 (0.979, 0.991)	<0.001
Male vs. female	1.16 (1.03, 1.29)	0.01	1.19 (1.05, 1.36)	0.007
Black vs. others	1.12 (1.01, 1.25)	0.04	1.02 (0.90, 1.16)	0.74
BMI	0.997 (0.987, 1.007)	0.60	-	
Diabetes vs. others	0.88 (0.79, 0.97)	0.01	0.92 (0.82, 1.04)	0.20
Preemptive vs. others	0.89 (0.75, 1.06)	0.20	-	
PRA >60 vs. 0-60%	1.14 (0.94, 1.38)	0.19	-	
Transplant				
HLA DR mismatch				
0	1.00		1.00	
1	1.17 (0.96, 1.43)	0.13	1.25 (0.97, 1.62)	0.08
2	1.33 (1.09, 1.63)	0.004	1.49 (1.17, 1.91)	0.001
Cold ischemic time	1.006 (1.001, 1.011)	0.04	1.003 (0.997, 1.009)	0.35
Transplant period				
2011-2014	1.00		1.00	
2008-2010	1.05 (0.92, 1.20)	0.44	1.02 (0.88, 1.18)	0.82
2005-2007	1.40 (1.23, 1.59)	<0.001	1.45 (1.25, 1.68)	<0.001
Induction therapy				
Thymoglobulin	1.00		1.00	
Alemtuzumab	1.03 (0.88, 1.22)	0.69	1.02 (0.85, 1.23)	0.84
Basiliximab	1.52 (1.33, 1.75)	<0.001	1.74 (1.49, 2.03)	<0.001
Other induction	0.96 (0.77, 1.19)	0.70	0.96 (0.75, 1.22)	0.74
No induction	1.14 (0.98, 1.33)	0.10	1.10 (0.92, 1.32)	0.30

CI, confidence interval; HR, hazard ratio; DCD, Donor after cardiac death; HCV, hepatitis C virus; BMI, body mass index; PRA, panel reactive antibody; HLA, human leukocyte antigen

d) Delayed graft function

Factors	Unadjusted		Adjusted	
	OR (95% CI)	P-value	OR (95% CI)	P-value
Glomerulosclerosis >10% vs. 0-10%	1.10 (0.99, 1.23)	0.07	1.10 (0.98, 1.23)	0.11
Donor				
Age	0.981 (0.976, 0.987)	<0.001	-	
Male vs. female	1.30 (1.20, 1.41)	<0.001	1.17 (1.04, 1.32)	0.01
Black vs. others	1.04 (0.96, 1.14)	0.34	-	
Diabetes vs. others	1.04 (0.95, 1.13)	0.38	-	
Hypertension vs. others	1.16 (1.05, 1.28)	0.003	0.99 (0.89, 1.11)	0.91
BMI	1.023 (1.017, 1.028)	<0.001	1.020 (1.014, 1.027)	<0.001
Log serum creatinine	1.71 (1.56, 1.88)	<0.001	1.52 (1.36, 1.71)	<0.001
DCD vs. others	2.36 (2.07, 2.69)	<0.001	-	
HCV antibody (+ vs. -)	1.08 (0.92, 1.27)	0.36	-	
Cerebrovascular accident as a cause of death vs. others	0.87 (0.79, 0.95)	0.003	0.90 (0.80, 1.00)	0.05
Machine perfusion (yes vs. no)	0.73 (0.68, 0.79)	<0.001	-	
Recipient				
Age	0.992 (0.988, 0.996)	<0.001	0.998 (0.994, 1.003)	0.51
Male vs. female	1.36 (1.26, 1.48)	<0.001	1.35 (1.22, 1.48)	<0.001
Black vs. others	1.54 (1.43, 1.68)	<0.001	1.46 (1.33, 1.61)	<0.001
BMI	1.027 (1.020, 1.034)	<0.001	1.022 (1.014, 1.032)	<0.001
Diabetes vs. others	1.38 (1.27, 1.49)	<0.001	1.30 (1.19, 1.43)	<0.001
Preemptive vs. others	0.27 (0.22, 0.31)	<0.001	0.23 (0.18, 0.28)	<0.001
PRA >60 vs. 0-60%	1.04 (0.90, 1.21)	0.59	-	
Transplant				
HLA DR mismatch			-	
0	1.00			
1	1.01 (0.88, 1.17)	0.87		
2	1.05 (0.91, 1.20)	0.53		
Cold ischemic time	1.013 (1.009, 1.017)	<0.001	1.012 (1.007, 1.017)	<0.001
Transplant period			-	
2011-2014	1.00			
2008-2010	1.03 (0.93, 1.13)	0.59		
2005-2007	0.95 (0.86, 1.05)	0.32		
Induction therapy				
Thymoglobulin	1.00		1.00	
Alemtuzumab	0.81 (0.71, 0.91)	0.001	0.75 (0.65, 0.86)	<0.001
Basiliximab	0.99 (0.89, 1.10)	0.80	0.95 (0.84, 1.08)	0.44
Other induction	1.51 (1.30, 1.74)	<0.001	1.41 (0.83, 1.08)	<0.001
No induction	0.96 (0.85, 1.07)	0.44	0.95 (0.83, 1.08)	0.42

CI, confidence interval; HR, hazard ratio; DCD, Donor after cardiac death; HCV, hepatitis C virus; BMI, body mass index; PRA, panel reactive antibody; HLA, human leukocyte antigen

Figure S1 Procurement cohort

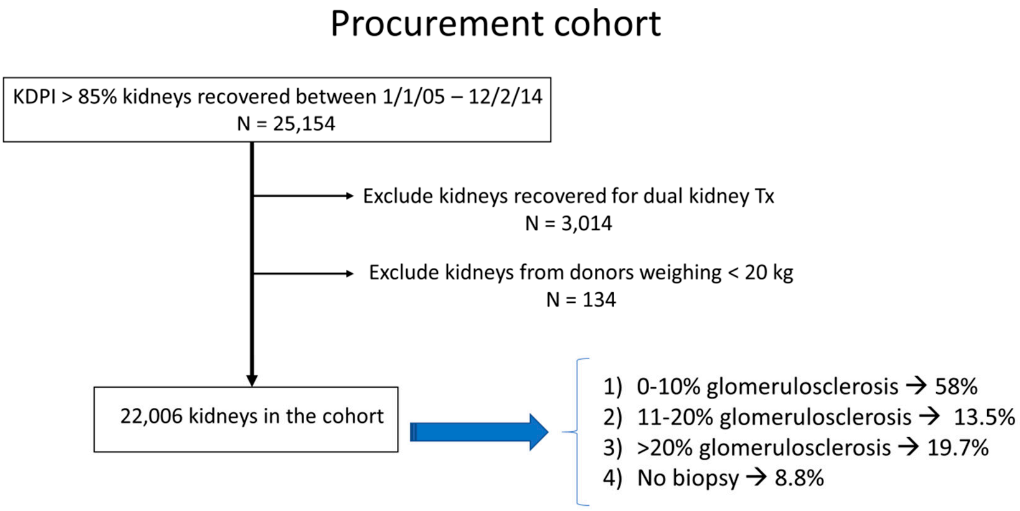

Figure S2 Kidney transplant recipient cohort

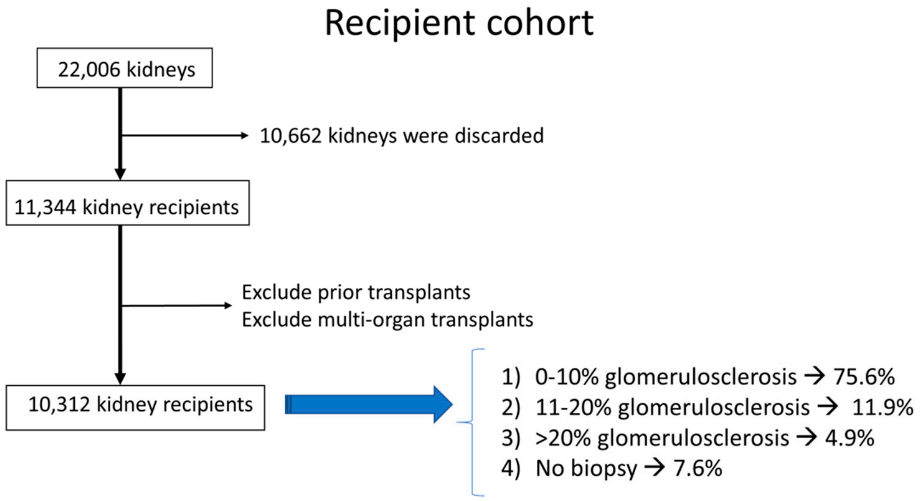

Supplement: Supplementary file 1 [file jcm-09-01469-s001.pdf]
